# Educational and accessible museums and cultural spaces

**DOI:** 10.1057/s41599-023-01563-8

**Published:** 2023-02-20

**Authors:** Ana Isabel González-Herrera, Andrea Betsabé Díaz-Herrera, Paula Hernández-Dionis, David Pérez-Jorge

**Affiliations:** Universidad de La Laguna, San Cristóbal de La Laguna, Spain

**Keywords:** Education, Science, technology and society

## Abstract

Offering access to culture and education to all citizens is a challenge nowadays, inclusive and accessible spaces are increasingly necessary if we really want to offer equal opportunities to all people regardless of their condition, physical or health. This systematic review study aims to investigate the situation of accessibility in museums and other cultural spaces as alternative learning spaces. It analyzes the historical evolution of cultural spaces as learning spaces and analyzes the reality of these spaces in terms of their accessibility conditions. For this purpose, an exhaustive search of documents was carried out between 2015 and 2021, following the PRISMA (Preferred Reporting Items for Systematic reviews and Meta-Analyses) statement, from the Web of Science (WOS), Scopus and Dialnet databases. After the analysis and application of selection criteria, a total of 17 documents were found that show the transformation of these cultural spaces, the improvement of their accessibility and adaptation to the new times. The need to offer cultural spaces for all is a challenge that must be consolidated as a social value.

## Introduction

In recent years, emphasis has been put on ensuring that people with disabilities or at risk of social exclusion can also have access to museums or heritage spaces because of their educational potential (McMillen & Alter, 2017; Martins, 2019; Hutchinson & Eardley, 2021). We understand, in this sense, that any space can provide opportunities for learning, especially when they are designed as spaces for learning.

In order to approach this study, concepts related to inclusion in cultural heritage and museums have been chosen. Through an exhaustive review of books and articles, interesting and outstanding information has been obtained on the reality of accessibility in these spaces. Bringing culture closer to society as a whole is a topic of great relevance, and investigating how it has been carried out in cultural spaces and museums is important, and given that no systematic reviews have been performed in this field, we consider this work to be innovative and relevant.

## Background

It is necessary to begin with the origin of cultural spaces. According to Martínez (2020), the first museums originated as spaces of restricted access that could only be accessed by certain groups. Cultural exclusion has been very common throughout history, and culture, an asset from which very few benefited. Making museums accessible and making culture available to all is an undeniable and necessary fact that requires the adoption of measures to promote an inclusive culture, regardless of the physical, sensory, intellectual, etc. condition of the people.

The term heritage is composed of a portion of the past that belongs to our present, it is configured from facts and decisions established by other people and that link us all.

Museums are spaces where all the value and culture of society is integrated; in this sense they must be able to undertake actions related to integration, accessibility and inclusion, aiming to provide the opportunity for all people to benefit from them regardless of their disability or condition (BOE, 1978). Museums are entities that imply a common benefit, where history is shared and culture is learned and acquired.

In the twentieth century, especially in recent decades, museums have been adapting significantly, establishing a more diverse and heterogeneous relationship with society, forming innovative, inclusive and global cultural spaces, with a new conception of culture and the way to access it. Step by step they have been consolidating their social function and assessing the experiences and needs of their audiences. Experts working in museums are increasingly aware of the diversity of their visitors, which leads to these cultural spaces becoming more accessible to the entire population, thus providing more and better opportunities to learn (Ibáñez, 2021). It is possible to talk about the museum as a space for social empowerment, with the integration of dialog and emotions, generating a progressive revolution of the museum and cultural spaces, as well as the increase in communication and heritage interpretation, which has progressively transformed the museum into an agent with social responsibility (Fernández Paradas et al., 2017).

According to Jiménez and García, (2020), in COVID-19 times, suddenly museums and cultural spaces were closed and the entire population had to stay at home. The projects, exhibitions, programs, activities, etc., that were being carried out by their employees, had to be canceled. This event posed new challenges, among which changing the paradigm of a classic museum model, with the need to modify its objectives, operation and organization, was a complex goal. This health crisis also had positive aspects, in this sense it gave us the opportunity to develop more inclusive and accessible actions, adapted to the whole society (Pérez-Jorge, et al., 2020; Jorge et al., 2022). The different cultural spaces, including museums, had to reinvent themselves to remain alive and accessible to citizens.

It is true that, throughout history, museums, art galleries, etc., have become formative spaces in the daily life of citizens. Cultural heritage has been developed and created so that the entire population can have access to it, however, it should be noted that there is still room for improvement and that not everything has been done.

The vision of creating accessible museums and cultural spaces involves transforming and modifying the way people think, since an inclusive and/or accessible space, implies improving infrastructures, including new methodologies for people with visual, hearing, motor, etc. disability, and also for other more vulnerable groups (Fernández Paradas et al., 2017). Making it accessible also entails modifying the cultural contents and the message they convey, as the way it is managed changes. Museums and cultural spaces must reach the whole of society and to do so they must adapt to their visitors.

Culture is an universal right, which must be considered from an inclusive perspective, without barriers or limitations that compromise its quality. Inclusive museums provide more specialized activities and actions for people with special needs, provide the opportunity for inclusion and offer options to welcome and address the needs of users in a process of adaptating spaces, materials and entrance limitations, in order to facilitate their full social inclusion.

On the other hand, cultural inclusion can help young people and teenagers to establish their identity; it is heritage that should have a primary role, and therefore the offers of heritage education and museums themselves should be inclusive. To achieve an accessible museum it is important to have a more specialized training of cultural agents working in museums, the most appropriate would be to form groups that teach through cooperative work, and promote more flexible educational projects that could be adapted to the differences and needs of each person. The physical, sensory and cognitive accessibility of the facilities should be improved, since museums and cultural spaces should guarantee equity and equal conditions for all visitors. In this way, activities should be established for all groups that visit these places to improve their integration, rather than focusing on the groups that have more social visibility. For this, we must be sensitive, designing non-discriminatory actions that can ensure equal opportunities.

The institutions that show art are spaces where the rules of coexistence and adaptation to social demands must be complied with. The most interesting aspect of this study is to know the reality of the accessibility exhibited by museums and cultural heritage in order to claim the relevant role they play as educational spaces. It is necessary to establish inclusive and not only accessible cultural spaces, which are dedicated to all audiences and not only to people with specific needs. According to (Quero et al., 2021), museums have employed resources and methods to make these cultural spaces more accessible, integrating accessible “tactile tours” and similar workshops. They have also included Braille brochures of artworks that most of the time include tactile relief graphics, for blind and visually impaired people. Currently, the relief artworks have been developed with the use of 3D printing techniques, as an alternative to improve accessibility for people with visual impairment. They have also been equipped with infrastructures and resources, for those who have motor or displacement difficulties. However, in addition to material resources, as stated by Pablos González, Fontal Merillas (2020), the availability of personnel resources is necessary.

This study provides information on accessibility in museums and cultural spaces by analyzing their evolution from their origins to the present day. The objectives of the study, developed as a systematic review work, are presented, as well as the inclusion and exclusion criteria for the search and selection of sources. The methodology and the results, along with discussion and conclusions, are addressed in the following sections.

This study pursues the following objectives

- To learn about the reality of accessibility and inclusion in museums and cultural spaces from an inclusive perspective.

- To investigate the most important topics related to inclusion, accessibility and cultural heritage.

- To carry out a critical analysis of accessible museums and cultural heritage.

## Methodology

The Dialnet and Web of Science (WOS) databases were used for this study. The selected databases were considered for the appropriateness and affinity of the publications with the field of education. The document search was conducted for the period 2015–2021, aiming to consider the most updated and recent information. Concerning the type of documents, no restriction was applied, with the intention of obtaining the largest number of papers related to the research topic. During the search and review process, various types of documents were found, such as articles, books, dissertations, theses, etc. For this study, a total of 17 journal articles were finally selected. For the selection of the studies considered, the following inclusion and exclusion criteria were applied (Table 1).

**Table 1:** Inclusion and exclusion criteria.

Inclusion criteria	Exclusion criteria
Articles related to accessibility and inclusion in cultural environments.	Studies that are related to the accessibility of other spaces.
Articles published in Dialnet and WOS databases.	Documents indexed in non relevant databases.
Documents published in the last 6 years	Articles in languages other than those selected
Articles in English and Spanish	Documents older than 6 years
Documents in Open Access	Documents no Open Access

### Description of the sources of information consulted and search descriptors

Three databases were consulted, WOS and Scopus for articles in English and Dialnet for articles in Spanish.

The following descriptors were used to search for information: “accessibility”, “inclusion”, “museums”, “cultural heritage”, “inclusive museums”, “museums and disability”. These descriptors were combined based on the Boolean operators AND and OR.

The search was carried out in the WOS and Scopus databases, always using the topics in English. On the other hand, a search for keywords in Spanish was carried out in the Dialnet database.

The documents were selected after filtering the search, based on the established inclusion and exclusion criteria. Search 1 used combinations of the topics: (museums AND “cultural heritage”) AND education. Search 2 used the terms: (museums AND “cultural heritage”) AND education AND inclusion. For search 3, the descriptors: (museums AND “cultural heritage”) AND accessibility AND education were used and, finally, for search 4, the topics: (museums AND “cultural heritage”) AND accessibility AND inclusion were used.

On the other hand, this process was carried out in Spanish in the Dialnet database, where four searches were also carried out with different combinations. First, the descriptors: “Museums, cultural heritage and accessibility” were used for search 1. Second, search 2 was carried out with the topics: “Museums, cultural heritage, accessibility and education”. For search 3, the following terms were used: “Museums, cultural heritage, education and inclusion”. To conclude the, search 4 was carried out using the following descriptors: “Museums, cultural heritages, accessibility and inclusion”.

It should be noted that more results were found on the Dialnet platform after performing the searches and applying the inclusion and exclusion criteria, with respect to the WOS and Scopus databases.

### Document selection method

The method used to perform the appropriate selection of the documents was the following:

- Development of the search in the different databases, inquiring about the key topics.

- Application of inclusion and exclusion criteria to refine the results obtained from the initial searches.

- Extraction of documents using the bibliographic manager Mendeley and elimination of duplicates.

- Selection of documents after review of document titles and reading of abstracts and keywords.

- Complete reading and analysis of the finally selected documents.

## Results

### Search strategy

Following the procedure outlined above and applying the different inclusion criteria, several searches were carried out in the databases mentioned above, combining the various topics according to the corresponding bolean operators.

First, searches were conducted in the WOS database, in search 1 (museums AND “cultural heritage”) AND education, 432 documents were found in total; after filtering the range of years from 2015 to 2021, 349 articles were obtained. Subsequently, after applying the Open Access criterion, 104 articles were left. Search 2 (museums AND cultural heritage) AND education AND inclusion obtained a total of 23 documents, after applying the criterion of the most recent years, 22 documents were found, out of which 6 were in Open Access and finally 15 were articles. Afterwards, search 3 (museums AND “cultural heritage”) AND accessibility AND education was performed, in which 13 documents were found in total, however, after filtering the criterion of the years 2015–2021, 10 documents were left, 6 of them were in Open Access and 12 were articles.

Finally, we proceeded to search 4 (museums AND “cultural heritage”) AND accessibility AND inclusion, from which a total of 3 documents were obtained, after filtering the most recent years 2015–2021, 3 articles were found in Open Access.

On the other hand, four searches were conducted in the Dialnet database, in Spanish. In search 1: Museums and “cultural heritages” and accessibility, 92 documents were found, but after applying the criterion of the years 2015–2021, 77 documents were left, out of which 76 were journal articles and/or books. In search 2: Museums and “cultural heritages” and accessibility and education, 69 documents were obtained in total, but when filtering the search on the range of the recent years, 63 overall documents were found, 60 being journal articles and/or books. Regarding search 3; Museums, “cultural heritages”, education and inclusion, a total of 67 documents were reached, after applying the range filter between 2015–2021, 62 documents were found, 60 being journal articles and/or books.

To conclude, we proceeded to search 4; museums and “cultural heritages” and accessibility and inclusion, from which a total of 61 documents were obtained; after applying the filter of the range of years between 2010 and 2019, the number decreased to a total of 59, out of which 57 were book and/or journal articles.

After having performed the search procedure by combining the keywords using Booleans, both in Spanish and English, it was possible to select the search finally used. For the Web of Science database the combination was (museums AND “cultural heritage”) AND education AND inclusion”, for the Dialnet search the combination was that corresponding to search 2; museums and “cultural heritage” and accessibility and education.

From all the articles that met the criteria, the following indicators were extracted: author/s, year of publication, document type, purpose of the study, research design used, sample, definition/concept, topic of interest and primary results (see Table 2).

**Table 2:** Summary of selected documents.

Citation (author/authors)	Title	Year	Document type	Purpose	Design	Definition Accessible museums and cultural heritage	Subject of interest	Results
Martínez and Calderón (2016)	The accessibility of museums: visions and perspectives.	2016	Research paper	To investigate accessibility in museums as an everyday activity.	Qualitative	Museums have become an everyday activity for society.	Museums, cultural heritage, accessibility and education	The museum should not be considered as a container of objects or a place reserved for educated people; the museum should generate feelings and guarantee equality for the entire population.
Martínez-Carrillo et al., (2019)	Accessible tourism for all. Analysis of the degree of accessibility of the museums of Tétouan (Morocco).	2019	Research paper	To know the degree of accessibility in museums, particularly in Tétouan.	Qualitative/quantitative (mixed)	Museums and culture should be accessible to the whole society, since it is a universal right.	Museums, cultural heritage, accessibility and education	According to UNWTO, in 2017, international tourist arrivals increased compared to 2016 Tourism is a relevant aspect of the economy as it produces 10% of taxs
Chiscano and Jiménez-Zarco (2021)	Towards an Inclusive Museum Management Strategy. An Exploratory Study of Consumption Experience in Visitors with Disabilities. The Case of the CosmoCaixa Science Museum	2021	Research paper	To offer inclusive orientations for a socially excluded group (people with disabilities) in museums and cultural spaces.	Qualitative	It analyzes museums as cultural spaces accessible to all citizens, without any kind of limitations or barriers. In addition, it promotes inclusion within these spaces, especially for people with functional diversity.	(“Museum and cultural heritage”) and accesibility and inclusion	Three factors were identified as influencing the inclusive experience in museums: awareness of universal design access requirements, access to information resources, and hospitality for visitors with disabilities.
Fernández Paradas et al. (2017)	Historical education. Forgotten heritages and happiness in didactics.	2017	Research paper	To learn about the research of a group of teachers and professionals who seek to improve their practice and facilitate the teaching-learning process of students.	Qualitative/quantitative (mixed)	Museums are accessible entities adapted for the whole society, including minorities and/or groups at risk of social exclusion.	Museums, cultural heritage, accessibility and education	The authors concluded that museums should be spaces of conviviality that inquire about the participation of their visitors.
García-Sampedro and Gutiérrez Berciano (2018)	The museum as a multicultural and learning space: some inclusive experiences.	2018	Review article	The evolution of museums and cultural spaces is analyzed, and the participation of citizens in these spaces is also promoted.	Qualitative	Museums used to be institutions focused on displaying collections and works of art, but today they are entities with communicative and educational functions, focused on the demands of their users.	Museums, cultural heritage, accessibility and education	Spanish museums are gradually developing strategies to attract strategies to attract non-habitual groups such as the most vulnerable social classes, the disabled, groups at risk of social exclusion, etc. vulnerable social classes, the disabled, groups at risk of social exclusion, etc.
Cagigal (2017)	Museums as Mediators of Memory in the Digital Age	2017	Research paper	It studies how to incorporate new technologies and the media in museums and cultural heritage.	Qualitative	Museums have been incorporating new technologies and media in order to include more attractive spaces for their visitors.	Museums, cultural heritage, accessibility and education	The author concluded that museums and the Internet are communicative media but that there is an advantage in these institutions since they have a dialogical and analytical balance.
Delgado and Oliva (2020)	Education and communication of heritage	2020	Research paper	Creativity in museums and cultural heritage is studied.	Qualitative	Cultural heritages and museums have been modifying their functions, focusing on the dissemination of knowledge and showing the collections to their users.	Museums, cultural heritage, accessibility and education	Education in museums is established as a field that is sustained from the practical field and that currently has very little theoretical consideration.
Melgar and Elisondo (2017)	Museos y Educación	2017	Research paper	To provide information about museums and educational contexts for a better understanding, a relationship between education and museums is established.	Qualitative	Museums as a space for training and learning.	Museums, cultural heritage, accessibility and education	Subjects must be willing to learn, and museums will be those spaces that provide such opportunities.
Mendieta Vargas (2015)	Inclusive museums: to see and not to touch?	2015	Research paper	Information about changes in the concept of disability and promotion of inclusive cultural spaces.	Qualitative	All people (including minorities) have the right to culture, so no one should be excluded from it.	Museums, cultural heritage, accessibility and education	This study concludes that museums should be spaces for all people, from people with disabilities to groups at risk of social exclusion.
Kosmas et al. (2020)	Enhancing accesibility in cultural heritage environments: considerations for social computing	2019	Research paper	Analyzes the different technological advances with respect to accessibility to culture and its corresponding spaces.	Qualitative	Technological advances have been incorporated to improve access to cultural heritage sites for people with functional diversity.	(“Museum and cultural heritage”) and accesibility and inclusion	The authors concluded that as a general rule, the requirements for an inclusive museum are not met, but they also detail that cultural institutions must be accessible, so work is being done to achieve this.
Sanz Simón et al. (2017)	Pedagogical museums as spaces for inclusion. A case study: The “Manuel Bartolomé Cossío” Museum/Laboratory for the History of Education.	2017	Research paper	Project based on people with autism spectrum disorder (ASD), for their inclusion in educational museums.	Qualitative	Educational museums should be places of inclusion for all people with disabilities, specifically for people with ASD.	Museums, cultural heritage, accessibility and education	New functions and areas of work are proposed to develop this minority group..
Uribe (2016)	Museums: Spaces to encourage knowledge and dissertations on the past?	2016	Research paper	Reflection on the role of the museum and its corresponding functions The museum as a reproduction of ideas	Qualitative	Museums were created through two historical periods, collecting and enlightenment.	Museums, cultural heritage, accessibility and education	The author proposed education in museums and their spaces as a liberating practice.
Taylor (2017)	From Systemic Exclusion to Systemic Inclusion: A Critical Look at Museums	2017	Dissemination article	Need to promote inclusion in cultural spaces and museums to guarantee culture for all citizens.	Qualitative/quantitative (mixed)	Systems thinking establishes the museum as institutions as a whole and not as separate entities.	(“Museum and cultural heritage”) and accesibility and inclusion	Through a survey some data was collected in which 61% people felt that MNHS has a culture of respect, while 31% felt it uses inclusive policies and practices.
Jiménez and García (2020)	Museums for the new times: nature from different points of view	2020	Research paper	Analysis on cultural spaces due to global pandemic and confinement looking for alternatives	Qualitative/quantitative (mixed)	The virtual museum as an alternative to expand culture in times of confinement after a global pandemic	Museums, cultural heritage, accessibility and education	Cultural heritage is still not within the reach of all citizens, only 49% of the population visits them.
Pisoni et al. (2021)	Human- Centered Artificial Intelligence for Designing Accesible Cultural Heritage	2021	Review article	Fostering technology to create accessible experiences in museums and other cultural heritage sites	Qualitative/quantitative (mixed)	Cultural heritages offer opportunities and challenges for the advancement of technological tools with the objective of being accessible to the public at large.	(“Museum and cultural heritage”) and accesibility and inclusion	Technology is being expanded to provide inclusive and accessible experiences
Spence (2020)	Scenting the Anosmic Cube: On the use of Ambient Scent in the Context of the Art Gallery or Museum	2020	Review article	To enhance the multisensory experience of visitors to art galleries and museums.	Qualitative/quantitative (mixed)	Most museums and galleries need to become more accommodating to their visually impaired visitors and thus create more engaging experiences	(“Museum and cultural heritage”) and accesibility and inclusion	Some people have considered establishing scents in art exhibits and museums to enhance the multisensory experience of their visitors.
Quero et al. (2021)	Accesible Visual Artworks for Blind and Visually Impaired People: Comparing a Multimodal Approach with Tactile Graphics	2021	Research paper	Study on accessible museums and cultural heritage looking for alternatives for the visually impaired and hearing impaired.	Qualitative/quantitative (mixed)	Museums and other cultural heritages are exploring more alternatives to achieve accessible and inclusive spaces, such as interactive guides for people with functional diversity.	(“Museum and cultural heritage”) and accesibility and inclusion	The study focused on people with visual and hearing impairments and 29.13% of them turned out to be visually and hearing impaired.

**Fig. 1:**
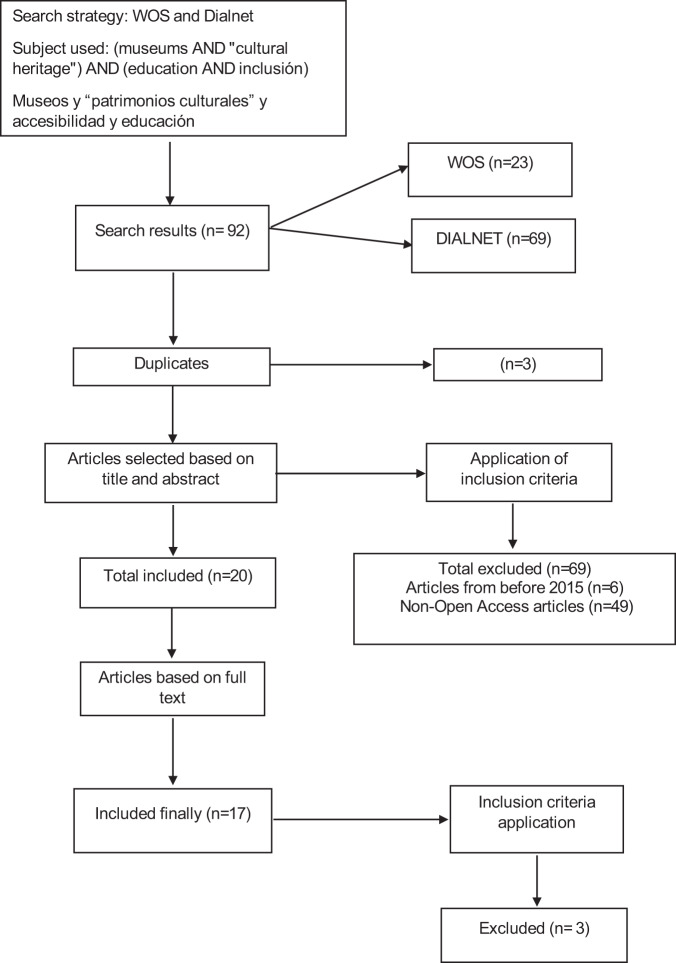
Flow chart document.

Given that accessibility in museums and cultural heritages is a relevant topic today, the articles reviewed were published between 2015 and 2021, as already explained. From these articles and based on the research methodology they followed, 10 were qualitative studies and 7 were mixed studies with qualitative and quantitative methodology. The final scheme of selected and excluded sources was established as reflected in Fig. 1.

### Analysis of the selected sources

According to Melgar, Elisondo (2017), museums began to be entities classified in non-formal education for conservation, research, training and leisure purposes, although, as they rightly point out these spaces have been criticized for being considered elitist, not very dynamic and boring. However, this conception changed in the mid-twentieth century, considering museums as social change agents. These institutions ceased to be spaces of conservation and exhibition and became places of communication with society.

García-Sampedro and Gutiérrez Berciano (2018) talk about how museums were considered public entities that responded to certain policies, with the aim of facilitating users simply to contemplate the works made. However, nowadays the functions have changed, and museums have become more flexible institutions in terms of cultural policies and adapt themselves to the needs of their visitors. New concepts such as “new museology”, “critical museology” and/or “didactic museum perspectives” have emerged (Martínez et al., 2017). These authors highlight the work of the museum today, these spaces are not only based on the maintenance of the collections on display, but also encompass functions such as training and communication within these spaces, to promote knowledge. Cultural and artistic activities are carried out in these spaces, including theater, dance, training, seminars, workshops, and in many cases, they have libraries or study rooms. The museum is considered as a point of reference of the cultural field to which all citizens can access.

Cagigal (2017) highlights that museums have been changing due to the influence of the digital era, specifically due to social networks and the large amount of information found on the internet (big data), since they share two aspects: “memory and social communication”. In addition, through technologies these spaces offer more means of information gathering and exhibition.

Ponce-Delgado and Oliva (2020) address the concept of cultural heritage and how it has been modified throughout history, according to the historical period and the changes that have occurred. Cultural heritage should promote the conservation of the set of objects, and scientific findings, but it should also share knowledge and exhibit collections, in order to promote culture and knowledge.

Martínez and Calderón (2016) address the accessibility of museums and inquire into the evolution of museums and cultural heritages, as inclusive and accessible spaces. They pointed out that the concept of “museum” has been modified, becoming spaces for learning, knowledge, and training, and not as exhibition places without any educational purpose. They consider that the museum should be a place accessible to all people, regardless of their condition and guaranteeing accessible spaces without barriers.

Along the same lines, the study by Martínez et al. (2017) points out that cultural spaces should be places within the reach of all citizens, without any type of discrimination, and without limitations. These places of leisure and learning should be adapted to all types of users, offering specific activities for people with special needs, with the aim of bringing culture closer to the whole society and fostering its inclusion. It is necessary to change the contents on display, the message they convey, the perspective of the employees who work there, etc., in response to the demands of new users.

As Chiscano and Jiménez-Zarco (2021) point out, the idea of inclusion in museums and cultural spaces is an advance in our history. They should be places of pedagogical use, in which activities and/or actions are carried out for all people, including people with more difficulties. They point out that they should offer more activities for people with disabilities, claiming the need to turn them into true inclusive spaces.

Fernández Paradas et al. (2017) estimate that museums should be considered as institutions with social responsibility. Museums have been modified themselves, providing a more accessible, inclusive, more flexible place, with more “open” ideologies. The authors point out that not all museums act in the same way, however, the primary commitment of museums should be based on social engagement to educate all citizens.

## Discussion

Museums and cultural heritages have been modified from past times to the present, they have become public places, where the needs and interests of their visitors must be addressed, since they offer the opportunity to learn and be trained in other educational contexts. Education is a fundamental right of people and should be available to all individuals, without any discrimination or differential circumstances (Jorge and Hernández, 2012).

Museums were traditionally considered as public entities dedicated to the exhibition of artwork and collections, visitors had a specific profile, most were educated people, and interested in this field. Fortunately, the change of paradigm in terms of the scope to be covered by education, has favored these spaces to focus on the needs of their visitors and not exclusively on the events or historical facts they treasure. Citizens need to actively participate in the visits and learning experiences that these places can provide, in this sense, the visit itself has to become a moment of reunion and social communication (Fernández Paradas et al., 2017).

The meaning of “cultural heritage” has been changing and today it must be understood as a dynamic fact exposed and subject to changes in the learning experience. Museums teach, but also transform with the participation of citizens, in this sense we can understand that universal access is an opportunity to change and update our cultural spaces and museums (Martínez et al., 2019; Ponce-Delgado and Oliva, 2020).

In this sense and as stated by Martínez and Calderón (2016) museums are becoming increasingly accessible and inclusive public spaces, more sensitive towards the changes and improvements they have to undertake to be able to welcome any citizen, regardless of their physical characteristics, personal conditions, gender, race, culture, etc., They have to adapt their contents to the conditions and social changes of the society that sustains them and they have to become comfortable spaces that people want to approach and immerse themselves in. Hence, new forms of management, new functions and new objectives are required.

They must be places without physical or ideological barriers, providing physical, sensory, cognitive, etc., resources to promote better inclusion in culture and its spaces. These institutions should adapt to the demands and needs of the population, offering more activities in which any minority can be included. Martínez-Carrillo et al. (2019).

Museums have also adapted to the present and to new technologies, due to the large amount of information found on the internet and the advance of social networks and the media (Cagigal, 2017), so that, through technologies, these institutions have expanded the means of information collection and in turn the exhibition. The use of social networks has been a good resource to expand culture to the entire population, and thanks to websites, more information about these spaces can be found.

## Conclusion

After the systematic review carried out, we can conclude that it is necessary to reflect on how these cultural spaces have been evolving and transforming, their accessibility as well as their adaptation to the new times, needs and demands of the public. Museums and other cultural spaces have ceased to be elitist centers dedicated to very specific areas and purposes to become open centers of participation and communication where not only needs are met but also the concerns of a society that increasingly wants to know. Culture, as an important factor in the development of society, is a universal asset and right that must be provided without exclusion, one of its objectives being showing and teaching about the different areas it encompasses: history, music, painting and other arts. The need to make the cultural offer reaching the entire population without exclusion means that more and more efforts are being made and more resources are being devoted to break down barriers, not only physical and sensory, but also to capture the attention of all audiences regardless of their ability to understand. To achieve this, it has been necessary not only to adapt content but also to find the ways and means that best suit each need and concern.

Cultural heritage, and within it museums, are not static spaces, but evolve with time and social demands. They are spaces open to knowledge in an increasingly interactive and participatory manner that guarantees free access to the entire population regardless of their condition, cultural level and vulnerability. It is the responsibility of all public and private institutions to guarantee the preservation and dissemination of culture and heritage. It is vitally important to make every effort to create an inclusive culture in an increasingly diverse society, with unequal needs where respect, equity and positive recognition of diversity are encouraged.

### Limitations

After the completion of this work, we have been able to ascertain the difficulties in accessing some documents not published in Open Access. The multidisciplinary nature of the topic has made it possible to demonstrate the interest in an increasingly current and latent subject that is approached from very different perspectives (architecture, museology, etc.), which has made it difficult to search for the educational dimension and approach and the training possibilities that museums can offer to the general public.

## Data Availability

The datasets generated during and/or analyzed during the current study are available from the corresponding author on reasonable request.
